# Transfer Extreme Learning Machine with Output Weight Alignment

**DOI:** 10.1155/2021/6627765

**Published:** 2021-02-11

**Authors:** Shaofei Zang, Yuhu Cheng, Xuesong Wang, Yongyi Yan

**Affiliations:** ^1^Department of Information Engineering College, Henan University of Science and Technology, Luoyang 471000, China; ^2^Department of Information and Control Engineering College, China University of Mining and Technology, Xuzhou 221116, China

## Abstract

Extreme Learning Machine (ELM) as a fast and efficient neural network model in pattern recognition and machine learning will decline when the labeled training sample is insufficient. Transfer learning helps the target task to learn a reliable model by using plentiful labeled samples from the different but relevant domain. In this paper, we propose a supervised Extreme Learning Machine with knowledge transferability, called Transfer Extreme Learning Machine with Output Weight Alignment (TELM-OWA). Firstly, it reduces the distribution difference between domains by aligning the output weight matrix of the ELM trained by the labeled samples from the source and target domains. Secondly, the approximation between the interdomain ELM output weight matrix is added to the objective function to further realize the cross-domain transfer of knowledge. Thirdly, we consider the objective function as the least square problem and transform it into a standard ELM model to be efficiently solved. Finally, the effectiveness of the proposed algorithm is verified by classification experiments on 16 sets of image datasets and 6 sets of text datasets, and the result demonstrates the competitive performance of our method with respect to other ELM models and transfer learning approach.

## 1. Introduction

Neural networks for solving classification problems have been widely researched in recent years [1, 2], which has powerful nonlinear fitting and approximation capabilities. Extreme Learning Machine (ELM), as a Single-Layer Feedforward Network (SLFN), has been proven to be an effective and efficient algorithm for pattern classification and regression [3, 4]. It randomly generates the input weight and bias of the hidden layer without tuning and only updates the weight between the hidden layer and the output layer. With the regular least squares (or ridge regression) as prediction error, the output weight will be efficiently obtained in a closed form by Moore–Penrose generalized inverse [3]. As a result, it has the advantages of strong generalization ability and fast training speed, therefore, and it has been widely used in various applications, such as face recognition [5], brain-computer interfaces [6–9], hyperspectral image classification [10], and malware hunting [11].

Although the learning speed and generalization ability of ELM are of great significance, there do exist many disadvantages. To improve ELM, many algorithms have been put forward in both theories and applications. In response to the fact that the shortcoming of ELM can be highly affected by the random selection of the input weights and biases of SLFN, Eshtay et al. [12] proposed a new model that uses Competitive Swarm Optimizer (CSO) to optimize the values of the input weights and hidden neurons of ELM. For imbalance data classification, Raghuwanshi and Shukla [13] presented a novel SMOTE based Class-Specific Extreme Learning Machine (SMOTE-CSELM), a variant of Class-Specific Extreme Learning Machine (CS-ELM), which exploits the benefit of both the minority oversampling and the class-specific regularization and has more comparable computational complexity than the Weighted Extreme Learning Machine (WELM) [14]. In order to reduce storage space and test time, the Sparse Extreme Learning Machine (Sparse ELM) [15] and multilayer sparse Extreme Learning Machine [16] were proposed for classification. To overcome the bias problem of a single Extreme Learning Machine, Voting based Extreme Learning Machine (V-ELM) [17, 18] and AdaBoost Extreme Learning Machine [19–21] are proposed to reduce the risk of selecting the wrong model by aggregating all candidate models. Moreover, some semisupervised ELM [22–25] and unsupervised ELM [26–28] algorithms were designed to utilize the large number of existing unlabeled samples for improving the performance of ELM and clustering. However, the above models are obtained under a typical assumption that the training and testing data are sampled from the identical distribution [29] and it may not always hold in many real worlds, yet the performance of ELM will degrade as a result of lacking sufficient samples with the same distribution for training model, and labeling samples are very expensive and costly [30].

Domain adaptation [31–33], as an important branch of transfer learning, solves the above problems with the help of the knowledge from the source domain which is different from but related to the target domain and resolves the inconsistency of sample distribution between the source and target domains. Zhang and Zhang [34] extended ELM to handle domain adaptation problems with very few labeled guide samples in target domain and overcome the generalization disadvantages of ELM in multidomain application. Li et al. [35] proposed the TL-ELM (transfer learning-based ELM) which uses a small amount of labeled target sample and a large number of labeled source samples to construct a high-quality classifier. Motivated by the biological learning mechanism, an Adaptive ELM (AELM) algorithm [36] was put forward for transfer learning which introduced the manifold regularization term into ELM for image classification under deep convolutional feature and representation. AELM is semisupervised transfer learning because it requires labels in the target domain. Due to the difficulty of collecting labels, unsupervised methods are more desirable. Chen et al. [37] presented a transfer ELM framework to bridge the source domain parameters and the target domain parameters by a projection matrix, in which informative source domain features are selected for knowledge transfer and the L2, 1-norm was applied to the source parameters. Li [38] and Chen [39], respectively, proposed two unsupervised domain adaptation Extreme Learning Machines by minimizing the classification loss and applying the Maximum Mean Discrepancy (MMD) strategy on the prediction results. Among the above approaches, due to efficiently utilizing target label, supervised ELM for transfer learning is superior to unsupervised ones.

**Figure 1:**
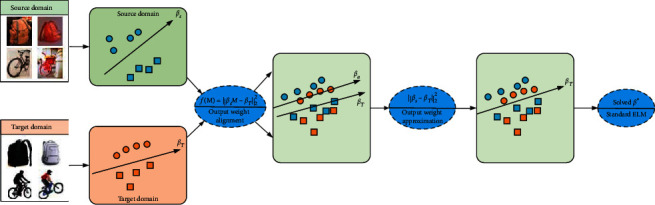
An illustration of TELM-OWA. (1) A mapping function that transforms the output weight of source ELM into one of target ELM is adopted to align the distribution between domains. (2) The output weight approximation constraint to prevent the negative transfer and realize the efficient transferring of knowledge across domains. (3) The objective function is transformed in standard ELM form to be solved.

In this paper, we focus on supervised transfer learning and propose a supervised ELM model with the ability of knowledge transfer, called Transfer Extreme Learning Machine with Output Weight Alignment (TELM-OWA), in which there are a small number of labeled target samples and a large number of labeled source samples to build a high-quality classification model. Firstly, it builds two ELM models utilizing labeled source and target samples. Secondly, we use a mapping function that transforms the output weight of source ELM into one of target ELM to align the distribution between the domains. Thirdly, a regularization constraint for the approximation between the interdomain ELM output weight matrices is added into the objective function to improve the cross-domain transfer of knowledge. Finally, we transform the objective function into a standard ELM form to solve and classify. Our approach is illustrated in Figure 1. Extensive experiments have been conducted on 16 sets of image datasets and 6 sets of text datasets and demonstrated significant advantages of our method over traditional ELM and state-of-the-art transfer learning methods.

The main contributions of this paper are as follows: (1) An idea of subspace alignment is adopted to reduce the distribution discrepancy between domains. (2) We apply the approximation constraint between the interdomain ELM output weight matrices to realize the efficient transferring of knowledge across domains. (3) The objective function is solved in standard ELM form, which is efficient and easy to understand. (4) Our proposed method performs image classification experiments on object recognition and text datasets. The results verify its effectiveness and advantage.

The remainder of this paper is as follows: in Section 2, we briefly introduce domain adaptation and ELM. In Section 3, we present TELM-OWA. In Section 4, the experiment and analysis to verify the validity of TELM-OWA are illustrated. Finally, Section 5 is the conclusion of the paper.

## 2. Related Work

### 2.1. Domain Adaptation

Transfer learning aims to learn a classifier for the target domain by leveraging knowledge from one or multiple well-labeled source domains. But if the source and target domains contain large different distribution data, its performance will be affected. In transfer learning, domain adaptation accelerates the cross-domain transfer of knowledge by minimizing the discrepancy between domains. According to “how to correct interdomain distribution mismatch,” domain adaptation can be roughly divided into three categories: sample weighting, subspace and manifold alignment, and statistical distribution alignment [33].

*Sample weighting* methods weigh each sample from the source domain to better match the target domain distribution and minimize the distribution divergence between two domains [40, 41], in which the estimation of the weights from the source samples is a key to this technique. The most classic sample-based transfer algorithm is TrAdaBoost proposed by Dai et al. [42]. It expands the AdaBoost algorithm and applies boosting technology to weigh the source and the target samples. Many algorithms are put forward to extend TrAdaBoost, such as DTrAdaBoost [43], Multisource-TrAdaboost (MTrA), and Task-TrAdaboost (TTrA) [44], Multi-Source Tri-Training Transfer Learning (MST3L) [45].

*Subspace and manifold alignment* methods try to align the subspace or manifold representations to preserve some important properties of data and simultaneously reduce the distribution discrepancy across domains. Subspace alignment (SA) [46–48] first projects the source and target samples into subspaces, respectively, and then functions a linear mapping to align the source subspace with the target ones and reduce cross-domain distribution difference for knowledge transfer.

*Statistical distribution adaptation* methods aim to explicitly evaluate and minimize the divergence of statistical distributions between the source and target domains to reduce the difference in the marginal distribution, conditional distribution, or both. To achieve this purpose, many statistical distances, such as Maximum Mean Discrepancy (MMD) [49], Bregman divergence [50], and KL divergence [51], are proposed for domain adaptation. Transfer Component Analysis (TCA) [52], Joint Distribution Analysis (JDA) [53], Weighted Maximum Mean Discrepancy (WMMD) [54], Transfer Subspace Learning (TSL) [55], and so forth are proposed to simultaneously tackle feature mapping, adaptation, and classification.

### 2.2. Extreme Learning Machine (ELM)

ELM is a fast learning algorithm for the single hidden layer neural network. Compared with the traditional neural network learning, it has two characteristics: (1) hidden layer parameters (i.e., input weights and the biases) can be randomly initialized. (2) The output layer weight can be solved as the least squares problem. As a result, ELM has a faster learning speed and more excellent generalization performance than traditional learning algorithms while guaranteeing higher accuracy.

**Figure 2:**
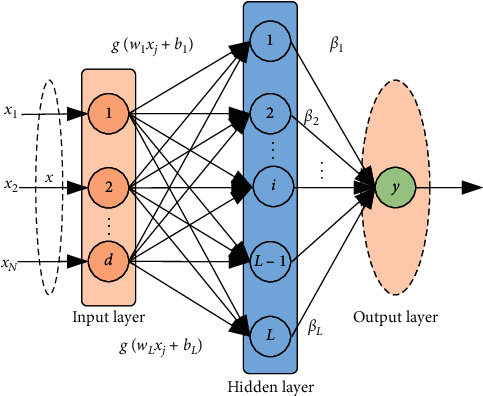
Basic structure of ELM.

Suppose giving a training dataset {(*x*_*i*_, *y*_*i*_)}_*i*=1_^*N*^ with *N* samples, where *y*_*i*_ ∈ *R*^*C*×1^ is the label corresponding to **x**_*i*_, and *C* is the number of categories. The structure of the ELM is shown in Figure 2.

In Figure 2, *x*_*i*_ is the input sample, *w*_*i*_ is the input layer weight, *b*_*i*_ is the hidden layer bias, *g*(*x*) is the nonlinear activation function, *L* is the number of nodes in the hidden layer, and *β*_*i*_ is the hidden layer output weight. The goal of ELM is to solve the optimal output weight *β*^*∗*^ by minimizing the sum of the squared loss function of the prediction error. The objective function is as follows:

(1) $$\begin{array}{c} \underset{\beta }{\min}\frac{1}{2}{\left\|\beta \right\|}^{2}+\frac{\theta }{2}\sum_{i=1}^{N}{\left\|e_{i}\right\|}^{2}\\ s.t.\;g\left(x_{i}\right)\beta =y_{i}^{T}-e_{i}^{T},i=1,\ldots ,N. \end{array}$$

In the previous equation, the first term is a regular term to prevent model overfitting, **e**_*i*_ is the error vector corresponding to the *i*-th sample, and *θ* is the tradeoff coefficient between the training error and the regular term.

Adding the constraint term to the objective function yields

(2) $$\begin{array}{c} \underset{\beta }{\min}L_{\text{ELM}}=\frac{1}{2}{\left\|\beta \right\|}^{2}+\frac{\theta }{2}{\left\|Y-H\beta \right\|}^{2}, \end{array}$$

where **H**=[*g*(**x**_*i*_)^*T*^,…,*g*(**x**_*N*_)^*T*^]^*T*^, $\beta =\left[\begin{array}{c} \beta_{1}\\ \vdots \\ \beta_{L} \end{array}\right]$, $Y=\left[\begin{array}{c} y_{1}\\ \vdots \\ y_{N} \end{array}\right]$.

The objective function is considered as a ridge regression or a regular least square problem. By setting the gradient of the objective function with respect to *β* to zero, we have

(3) $$\begin{array}{c} \nabla L_{\text{ELM}}=\beta +\theta H^{T}\left(Y-H\beta \right)=0. \end{array}$$

There are two cases in the process of solving *β*. If *N* ≤ *L*, equation (3) is overdetermined [20]; the optimal solution is

(4) $$\begin{array}{c} \beta^{*}=\left(H^{T}H+\frac{I_{L}}{\theta }\right)H^{T}Y, \end{array}$$

where **I**_*L*_ is a *L*-dimensional unit matrix.

If *N* ≥ *L*, equation (3) is underdetermined [23]; the optimal solution is

(5) $$\begin{array}{c} \beta^{*}=H^{T}\left(H^{T}H+\frac{I_{N}}{\theta }\right)Y, \end{array}$$

where **I**_*N*_ is an *N*-dimensional unit matrix.

In the classification task, given a sample *x*_*Te*_ to be tested, the classification result can be obtained:

(6) $$\begin{array}{c} y_{Te}=\left\{\begin{array}{cc} \text{sign}\left(h_{Te}^{T}\beta^{*}\right), & \text{for binary classification},\\ \arg\max\left(h_{Te}^{T}\beta^{*}\right), & \text{for multiclassification,} \end{array}\right) \end{array}$$

where **h**_*Te*_=*g*(**x**_*Te*_).

## 3. TELM-OWA

In the past few years, the theory and application of ELM have received extensive attention from scholars and great progress has been made in this field. However, when there are fewer training samples, the performance of ELM will decrease [34]. Transfer learning draws on relevant domain knowledge to improve the learning efficiency of tasks in the target domain [31]. Therefore, through transfer learning, the performance of ELM can be improved in the case of insufficient labeled samples.

In transfer learning, there are two different but related datasets: source domain **D**_*S*_={(**x**_*s*(*i*)_, **y**_*s*(*i*)_)}_*i*=1_^*n*_*S*_^ and target domain **D**_*T*_=**D**_*Tr*_∪**D**_*Te*_={(**x**_*T*(*j*)_, **y**_*T*(*j*)_)}_*j*=1_^*n*_*T*_^∪{**x**_*Te*(*k*)_}_*k*=1_^*n*_*Te*_^. **x**_*s*(*i*)_ and **y**_*s*(*i*)_ are the source domain sample and its label, respectively, and *n*_*S*_ is the number of **D**_*S*_ samples. Accordingly, **x**_*T*(*j*)_ ∈ **D**_*Tr*_ and **y**_*T*(*i*)_ ∈ **D**_*Tr*_ are the target labeled sample and its corresponding label, respectively, **x**_*Te*(*k*)_ ∈ **D**_*Te*_ is the target unlabeled sample, *n*_*T*_ and *n*_*Te*_ are the number of labeled and unlabeled samples in **D**_*T*_, and *n*_*T*_ ≪ *n*_*S*_. In this section, we hope to construct an ELM model using {(**x**_*T*(*j*)_, **y**_*T*(*j*)_)}_*j*=1_^*n*_*T*_^∪{(**x**_*s*(*i*)_, **y**_*s*(*i*)_)}_*i*=1_^*n*_*S*_^ to obtain high accuracy on {**x**_*Te*(*k*)_}_*k*=1_^*n*_*Te*_^.

### 3.1. Output Layer Weight Alignment

By using the source domain labeled samples and the target domain labeled samples, respectively, two ELM can be built as follows:

(7) $$\begin{array}{c} \underset{\beta_{S}}{\min}:\frac{1}{2}{\left\|\beta_{S}\right\|}^{2}+\frac{1}{2}{\left\|H_{S}\beta_{S}-Y_{S}\right\|}^{2},\\ \underset{\beta_{T}}{\min}:\frac{1}{2}{\left\|\beta_{T}\right\|}^{2}+\frac{1}{2}{\left\|H_{T}\beta_{T}-Y_{T}\right\|}^{2}, \end{array}$$

where **H**_*S*_ is the hidden layer output matrix of **D**_*S*_ and *β*_*S*_ is the output layer weight of the ELM obtained by **D**_*S*_ training. Accordingly, **H**_*T*_ is the output layer output matrix of **D**_*T*_ and *β*_*T*_ is the out-layer weight of the ELM obtained by {(**x**_*T*(*j*)_, **y**_*T*(*j*)_)}_*j*=1_^*n*_*T*_^ training. Due to the difference in the distribution between **D**_*S*_ and {(**x**_*T*(*j*)_, **y**_*T*(*j*)_)}_*j*=1_^*n*_*T*_^, it can be known that *β*_*S*_ ≠ *β*_*T*_. Inspired by the literature [46, 47], the transformation matrix **M** is used to align the output layer of ELM between the source domain and the target domain in order to achieve cross-domain knowledge transferring. The function is established as follows:

(8) $$\begin{array}{c} f\left(M\right)={\left\|\beta_{S}M-\beta_{T}\right\|}_{F}^{2}, \end{array}$$

where ‖·‖_*F*_^2^ is Frobenius mode. It can be known from the previous equation [43] that **M**^*∗*^=min*f*(**M**).

Since the Frobenius mode is invariant to the orthogonalization operation [46], equation (8) can be rewritten as

(9) $$\begin{array}{c} f\left(M\right)={\left\|\beta_{S}^{T}\beta_{S}M-\beta_{S}^{T}\beta_{T}\right\|}_{F}^{2}={\left\|M-\beta_{S}^{T}\beta_{T}\right\|}_{F}^{2}. \end{array}$$

**Figure 3:**
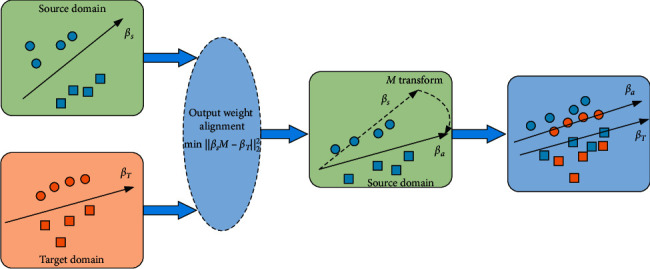
Illustration of Output Weight Alignment method.

For equation (9), we can conclude that the optimal **M***∗*=*β*_*S*_^*T*^*β*_*T*_. Therefore, *β*_*a*_=*β*_*S*_**M**=*β*_*S*_*β*_*S*_^*T*^*β*_*T*_ can be regarded as the output layer weight after the output layer of the source domain ELM model is aligned to the target domain, as shown in Figure 3.

### 3.2. Objective Function of TELM-OWA

In order to realize the transfer of the Extreme Learning Machine, the following objective function can be established to solve

(10) $$\begin{array}{c} J\left(\beta_{S},\beta_{T}\right)=\underset{\beta_{S},\beta_{T}}{\min}:\frac{1}{2}{\left\|\beta_{T}\right\|}^{2}+\frac{\lambda }{2}{\left\|H_{S}\beta_{S}-Y_{S}\right\|}^{2}+\frac{1}{2}{\left\|H_{T}\beta_{T}-Y_{T}\right\|}^{2}+\frac{\gamma }{2}{\left\|\beta_{T}-\beta_{S}\right\|}^{2}, \end{array}$$

where (*γ*/2)‖*β*_*T*_ − *β*_*S*_‖^2^ is a regular term for facilitating knowledge transfer and preventing negative transfer and *λ*, *γ* are the balance parameter.

To align the output layer of source ELM to target one, we replace *β*_*S*_ with *β*_*a*_ and substitute it into equation (10) to get

(11) $$\begin{array}{c} J\left(\beta_{a},B_{T}\right)=\underset{\beta_{a},\beta_{T}}{\min}\frac{1}{2}{\left\|\beta_{T}\right\|}^{2}+\frac{\lambda }{2}{\left\|H_{S}\beta_{a}-Y_{S}\right\|}^{2}+\frac{1}{2}{\left\|H_{T}\beta_{T}-Y_{T}\right\|}^{2}+\frac{\gamma }{2}{\left\|\beta_{T}-\beta_{a}\right\|}^{2}. \end{array}$$

Because of *β*_*a*_=*β*_*S*_*β*_*S*_^*T*^*β*_*T*_, equation (11) becomes

(12) $$\begin{array}{c} J\left(\beta_{S},\beta_{T}\right)=\underset{\beta_{S},\beta_{T}}{\min}\frac{1}{2}{\left\|\beta_{T}\right\|}^{2}+\frac{\lambda }{2}{\left\|H_{S}\beta_{S}\beta_{S}^{T}\beta_{T}-Y_{S}\right\|}^{2}+\frac{1}{2}{\left\|H_{T}\beta_{T}-Y_{T}\right\|}^{2}+\frac{\gamma }{2}{\left\|\beta_{T}-\beta_{S}\beta_{S}^{T}\beta_{T}\right\|}^{2}\\ =\underset{\beta_{S},\beta_{T}}{\min}\frac{1}{2}{\left\|\beta_{T}\right\|}^{2}+\frac{\lambda }{2}{\left\|H_{S}\beta_{S}\beta_{S}^{T}\beta_{T}-Y_{S}\right\|}^{2}+\frac{1}{2}{\left\|H_{T}\beta_{T}-Y_{T}\right\|}^{2}+\frac{\gamma }{2}{\left\|\left(I-\beta_{S}\beta_{S}^{T}\right)\beta_{T}\right\|}^{2}. \end{array}$$

Because ‖(**I** − *β*_*S*_*β*_*S*_^*T*^)*β*_*T*_‖^2^ ≤ ‖(**I** − *β*_*S*_*β*_*S*_^*T*^)‖^2^‖*β*_*T*_‖^2^, we change equation (12) into

(13) $$\begin{array}{c} J\left(\beta_{S},\beta_{T}\right)\\ =\underset{\beta_{S},\beta_{T}}{\min}\frac{1}{2}{\left\|\beta_{T}\right\|}^{2}+\frac{\lambda }{2}{\left\|H_{S}\beta_{S}\beta_{S}^{T}\beta_{T}-Y_{S}\right\|}^{2}+\frac{1}{2}{\left\|H_{T}\beta_{T}-Y_{T}\right\|}^{2}+\frac{\gamma }{2}{\left\|\left(I-\beta_{S}\beta_{S}^{T}\right)\right\|}^{2}{\left\|\beta_{T}\right\|}^{2}\\ =\underset{\beta_{S},\beta_{T}}{\min}\frac{1}{2}{\left\|\left(\begin{array}{c} \lambda H_{S}\beta_{S}\beta_{S}^{T}\\ H_{T} \end{array}\right)\beta_{T}-\left(\begin{array}{c} Y_{S}\\ Y_{T} \end{array}\right)\right\|}^{2}+\frac{\left(I+\gamma {\left(I-\beta_{S}\beta_{S}^{T}\right)}^{T}\left(I-\beta_{S}\beta_{S}^{T}\right)\right)}{2}{\left\|\beta_{T}\right\|}^{2}. \end{array}$$

Let $Q=\left(\begin{array}{c} \lambda H_{S}\beta_{S}\beta_{S}^{T}\\ H_{T} \end{array}\right)$，$T=\left(\begin{array}{c} Y_{S}\\ Y_{T} \end{array}\right)$，**A**=**I**+*γ*(**I** − *β*_*S*_*β*_*S*_^*T*^)^*T*^(**I** − *β*_*S*_*β*_*S*_^*T*^), and the objective function of TELM-OWA can be simplified as

(14) $$\begin{array}{c} J\left(\beta_{T}\right)=\underset{\beta_{T}}{\min}:\frac{1}{2}{\left\|Q\beta_{T}-T\right\|}^{2}+\frac{A}{2}{\left\|\beta_{T}\right\|}^{2}, \end{array}$$

and, then,

(15) $$\begin{array}{c} \beta_{T}^{*}=\left\{\begin{array}{cc} {\left(Q^{T}Q+A\right)}^{-1}Q^{T}T, & n>L,\;I\text{ in }A\text{ is an }L-\text{dimensional unit matrix},\\ Q^{T}{\left(QQ^{T}+A\right)}^{-1}T, & n\leq L,\;I\text{ in }A\;i\text{s an }n-\text{dimensional unit matrix}. \end{array}\right) \end{array}$$

After *β*_*T*_ is obtained with knowledge transferability, the test samples are classified by equation (6). A complete classification procedure of TELM-OWA is summarized in Algorithm 1.

### 3.3. Discussion

In order to improve the classification performance of ELM under transfer learning environment, we propose TELM-OWA and its objective function is equations (11) to (14) which can be seen as follows:

(1) Compared with the traditional ELM, TELM-OWA adopts ‖**H**_*S*_*β*_*a*_ − **Y**_*S*_‖^2^ to utilize the source domain knowledge to help the target ELM to obtain the optimal parameter *β*_*T*_^*∗*^ and also increases the fitness of *β*_*T*_^*∗*^ to the target domain data by ‖**H**_*T*_*β*_*T*_ − **Y**_*T*_‖^2^.
(2) DAELM-S proposed by Pan and Yang [34] also applies ‖**H**_*S*_*β*_*S*_ − **Y**_*S*_‖^2^ to help target task, in which the objective function is as follows:
  (16) $$\begin{array}{c} \min\frac{1}{2}{\left\|\beta_{S}\right\|}^{2}+\frac{C_{S}}{2}{\left\|H_{S}\beta_{S}-Y_{S}\right\|}^{2}+\frac{C_{T}}{2}{\left\|H_{T}\beta_{S}-Y_{T}\right\|}^{2}. \end{array}$$
Though DAELM-S uses ‖**H**_*S*_*β*_*S*_ − **Y**_*S*_‖^2^ to transfer the knowledge from the source domain and increases the fitness of *β*_*S*_ to source data, this decreases the fitness to the target domain comparing with TELM-OWA in which *β*_*a*_ is more approximated to *β*_*T*_ than *β*_*S*_ by applying a subspace alignment mechanism.
Therefore, ‖**H**_*S*_*β*_*a*_ − **Y**_*S*_‖^2^ can increase the fitness of *β*_*T*_^*∗*^ to target data more than ‖**H**_*S*_*β*_*S*_ − **Y**_*S*_‖^2^, and ‖*β*_*T*_ − *β*_*a*_‖^2^ can promote the transfer of knowledge across domains. As a result, TELM-OWA has stronger knowledge transfer capabilities than DAELM-S.
(3) Although DAELM-T proposed by Zhang et al. [34] uses ‖**H**_*Tu*_*β*_*T*_ − **H**_*Tu*_*β*_*S*_‖^2^ to promote the approximation of *β*_*S*_ and *β*_*T*_, the objective function is as follows:
  (17) $$\begin{array}{c} \min\frac{1}{2}{\left\|\beta_{T}\right\|}^{2}+\frac{C_{T}}{2}{\left\|H_{T}\beta_{T}-Y_{T}\right\|}^{2}+\frac{C_{Tu}}{2}{\left\|H_{Tu}\beta_{T}-H_{Tu}\beta_{S}\right\|}^{2}. \end{array}$$
However, ‖*β*_*T*_ − *β*_*a*_‖ < ‖*β*_*T*_ − *β*_*S*_‖ is obvious according to equation (9) and Figure 3. Therefore, TELM-OWA has a better knowledge transfer effect than DAELM-T.
(4) Because TELM-OWA and DAELM-T need to firstly solve *β*_*s*_ when solving the optimal parameter *β*_*T*_^*∗*^, therefore, compared with ELM and DAELM-S, TELM-OWA and DAELM-T have more computing complexity of *O*(*L*^3^), where *L* is the number of hidden layer nodes.
(5) In [37], PTELM also adopted Output Weight Alignment based on ELM for knowledge transfer. But there are two differences between PTELM and TELM-OWA. On one hand, PTELM is suitable for unsupervised transfer learning in which no target label is needed, but TELM-OWA is an supervised transfer learning algorithm requiring little target label. On the other hand, PTELM needs to solve the projection matrix for Output Weight Alignment and output weight adopting the coordinate descent method in alternatively optimizing manner. In TELM-OWA, output weight is only needed to be solved as the standard ELM.

## 4. Experiment and Analysis

To verify the validity of TELM-OWA, four different datasets, Office + Caltech object recognition, USPS and MNIST digital handwriting, MSRC and VOC2007 object recognition, Reuters-21578 text dataset, are used for classification experiments, where image and text datasets are described in Table 1. All the experiments are carried out on a PC with 8 GB memory and Windows 10 operating system. The algorithms are implemented in MATLAB 2017b. Each experiment is done 20 times, and the result is taken as average. The accuracy of each algorithm is evaluated by the accuracy rate and the formula is as follows:

(18) $$\begin{array}{c} \text{accuracy}=\frac{\text{correctly\_classified\_samples}}{\text{total\_samples}}\times 100\%. \end{array}$$

### 4.1. Dataset Description

- **USPS** **+** **MNIST**: both USPS and MNIST are image datasets that describe handwritten numbers. They are different but related, with a total of 10 digital categories. During the experiment, two sets of experimental data (USPS vs. MNIST, MNIST vs. USPS) were constructed as follows: 1800 images were randomly selected from USPS as source and target domain datasets, and correspondingly, 2000 samples were randomly selected from MNIST as the target domain and source domain datasets. All pictures in USPS and MNIST are uniformly transformed into pixels of 16 × 16, and each picture is changed into a grayscale image representing pixel points by gray values.
- **MSRC** **+** **VOC**: the MSRC dataset is provided by Microsoft Cambridge, which contains 18 categories for a total of 4323 images. The VOC2007 dataset contains 20 categories for a total of 5011 images. MSRC and VOC2007 have distinct but different distributions. The MSRC is evaluated with standard images as benchmark data. VOC2007 is built freely with images from web albums. They share the following 6 semantic categories: airplanes, birds, cows, family cars, sheep, and bicycles. The transfer learning dataset MSRC versus VOC is constructed, in which 1269 subpictures are selected as the source domain dataset from the MSRC dataset, and 1530 subpictures are selected from the VOC2007 dataset as the target domain dataset. Then, we exchange the source and target domain to build a new set of transfer learning datasets VOC versus MSRC. We convert all the images into 0∼256 gray pixels and extract 240 dimensions as the spatial dimension of the sample.
- **Office** **+** **Caltech**: Office is a common dataset for visual cross-domain learning, with 3 realistic aggregated item datasets: Amazon (downloaded by online trading website), Webcam (photographed by low-resolution webcam), and DSLR (photographed by digital SLR high-resolution camera). This dataset contains 4,652 images in 31 categories. Caltech is also a standard dataset commonly used for target recognition. It contains 30,607 images in 256 categories. The Office + Caltech dataset released by Gong [56] contains four fields C (Caltech-256), A (Amazon), W (Webcam), and D (DSLR) in the 10 common classes. During the experiment, two different fields are randomly selected as the source and target domain datasets and 12 cross-domain target datasets can be constructed, namely, C⟶A, C⟶W, C⟶D, ..., and D⟶W.
- **Reuters-21578**: the Reuters-21578 text dataset, which is a common dataset for text categorization, containing 21,577 news articles from Reuters in 1987 that were manually labeled by Reuters with 5 classes including “exchanges,” “orgs,” “people,” “places,” and “topics.” 5 classes are divided into multiple major classes and subclasses. The three largest classes shown in Table 1 are “orgs,” “people,” and “place,” which can construct 6 cross-domain text classification tasks as orgs versus people, people versus orgs, orgs versus place, place versus orgs, people versus place, and place versus people. The article conducted a more intensive evaluation on 6 classification tasks.

### 4.2. Experimental Results and Analysis

We compared the proposed algorithm with some classifiers for evaluating the performance.

#### 4.2.1. Classifier of Nontransfer Learning

- 1NN: *k* nearest neighbor classifier with one nearest neighbor.
- SVM: support vector machine with the linear kernel.
- ELM: Standard Extreme Learning Machine.
- SSELM [23]: ELM with graph regularization term for semisupervised learning.

#### 4.2.2. Classifier for Transfer Learning

- TCA [52] + 1NN: classifier is built by combining TCA with 1NN for the classification task of transfer learning.
- TCA [52] + SVM: classifier is built by combining TCA with SVM for the classification task of transfer learning.
- JDA [53] + 1NN: classifier is built by combining JDA with 1NN for the classification task of transfer learning.
- JDA [53] + SVM: classifier is built by combining JDA with SVM for the classification task of transfer learning.
- DAELM-S [34]: ELM trained using a number of source labeled data and a limited number of target labeled data for domain adaptation.
- DAELM-T [34]: ELM trained using a limited number of target labeled data and numerous target unlabeled data to approximate the prediction from ELM trained using source data; ARRLS [57]: a general transfer learning framework referred to adaptation regularization based transfer learning using squared loss.
- TELM-OWA: we proposed a supervised classifier called Transfer Extreme Learning Machine with Output Weight Alignment.

In the experiment, we set the SVM penalty parameter belonging to {0.1, 0.5, 1,5,10,50,100}, and the penalty parameter *θ* ∈ [0.001, 0.1] in ELM, SSELM, DAELM_S, DAELM_T, and TELM-OWA. TCA and JDA are feature transfer algorithms, which are combined with PCA to achieve the extraction of shared feature subspace based on MMD. In the above feature transfer algorithms, the dimension of the feature subspace is 100. The value range of the balanced-constraint parameter of the projection matrix in TCA and JDA algorithm is [0.1, 1]; ARRLS algorithm combines JDA with structural risk minimization and graph regular terms to improve knowledge transfer effect. Its parameters are set according to [57].

Among them, in each dataset, 20% of the total number of target domain samples are randomly selected as a small number of labeled samples and are used as test sample sets together with source domain samples. In1NN, SVM, ELM, SSELM, TCA + (1NN, SVM), JDA + (1NN, SVM), and ARRLS, the labeled samples from the source and target domain are used together to train the classifier.  Table 2 shows the classification results of the algorithms on the image and text datasets.

**Figure 4:**
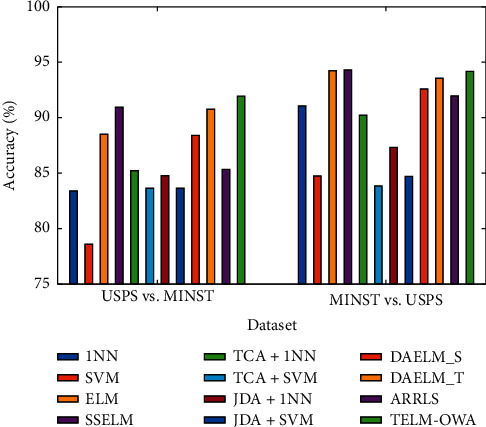
Classification accuracy of different algorithms on USPS + MNIST dataset.

**Figure 5:**
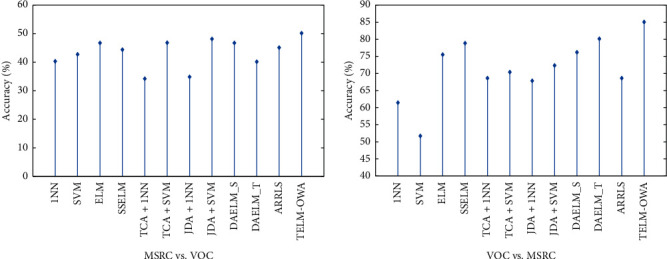
Classification accuracy of different algorithms on MSRC + VOC dataset.

**Figure 6:**
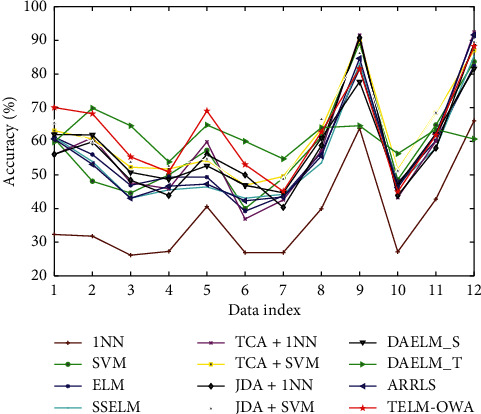
Classification accuracy of different algorithms.

**Figure 7:**
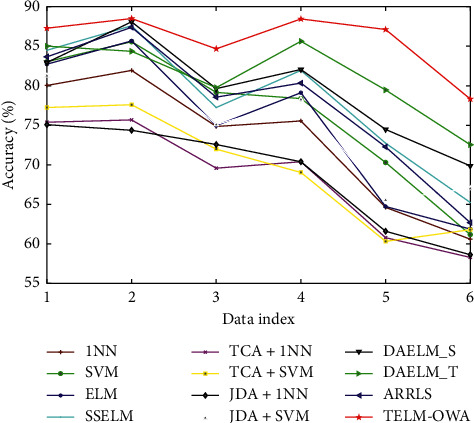
Classification accuracy of different algorithms. On Office + Caltech dataset on Reuters-21578 dataset.

The classification results from Table 2 and Figures 4–7 prove the following: (1) first, the average accuracy of TELM-OWA across 22 tasks is 72.13%, which is obvious that TELM-OWA outperforms other methods on most tasks. (2) TELM-OWA outperforms DAELM_S, DAELM_T, indicating the superiority of Output Weight Alignment and ‖*β*_*T*_ − *β*_*S*_‖^2^, which promotes the transfer of knowledge across domains. (3) TELM-OWA DAELM_S and DAELM_T achieve good results compared to other most algorithms. It shows that ELM with the ability of knowledge transfer has a high performance for transfer learning. (4) The standard machine learning methods, that is, 1NN, SVM, and ELM, suffer from the domain shift problem; thus, they could obtain an unsatisfied performance. But ELM gains more significant performance than 1NN and SVM because of its good fitness and generality to data. (5) The semisupervised method SSELM performs better than ELM by exploring the geometry property of domain, but worse than TELM-OWA, DAELM_S, and DAELM_T without considering domain shift problem. (6) Due to the lower accuracy of 1NN, TCA + 1NN and JDA + 1NN are worse than SVM, ELM, TCA + SVM, and JDA + SVM but higher than 1NN. (7) The accuracy of the feature extraction algorithm with transfer capability, such as TCA + SVM and JDA + SVM, is higher than SVM, which is similar to 1NN as a classifier, indicating the importance of feature transfer learning in the case of few or not the same distribution samples. (8) The accuracy of JDA + 1NN and JDA + SVM is generally higher than TCA + 1NN and TCA + SVM, which indicates the superiority of reducing the marginal and conditional distribution discrepancy at the same time. (9) ARRLS generally outperforms all baseline methods by minimizing the difference between both marginal and conditional distributions, meanwhile preserving the manifold consistency.

The computer time-consuming algorithms of 1NN, SVM, ELM, SSELM, TCA + 1NN, TCA + SVM, JDA + 1NN, JDA + SVM, DAELM_S, DAELM_T, ARRLS, and TELM-OWA on MNIST versus USPS datasets are investigated, respectively, as shown in Table 3. The following can be seen: (1) the time cost of method based ELM is less than other algorithms except for 1NN, indicating that the speed of ELM is superior to the other. (2) TELM-OWA consume more time than ELM, SSELM, DAELM_S, and DAELM_T, because it needs to firstly solve *β*_*S*_^*∗*^ and then obtain *β*_*T*_^*∗*^. (3) SSELM consumes more time than ELM, SSELM, DAELM_S, and DAELM_T, because it needs to construct Laplace graph matric and then obtain *β*_*T*_^*∗*^. (4) The classifiers with feature extraction consume more time than the standard classifier according to it. (5) The cost time of the method based on SVM is higher than other algorithms. (6) JDA + 1NN and JDA + SVM apply an iterative manner to refine the pseudo label from target domains, so their time cost is higher than TCA + 1NN and TCA + SVM.

**Figure 8:**
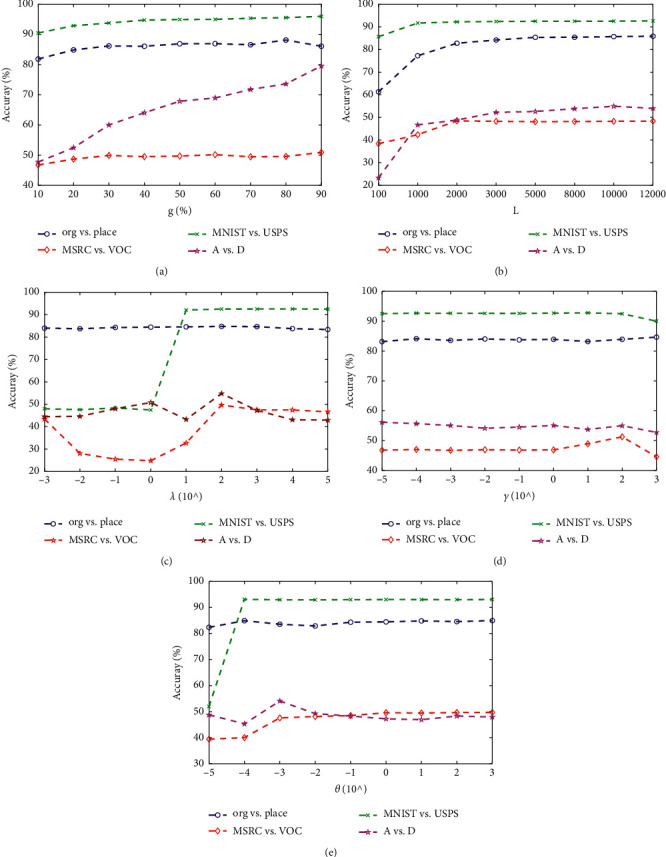
Effect of number of labeled target samples (g), number of hidden layer nodes, and parameters *λ*, *γ*, and *θ* on accuracy.

Moreover, in Tables 2–3 and Figures 4–7 we can see the following: (1) TELM-OWA, as an extension of ELM in transfer learning, also has faster learning speed and higher accuracy than other non-ELM methods, because it maintains the advantages of the good fitness of neural network and ridge regression model with a closed-form solution. (2) Although TELM-OWA has higher accuracy than ELM, SSELM, DAELM_S, and DAELM_T, it also has more learning time. When *L* > 2000, if the number of hidden-layer nodes is reduced, its learning speed will improve but its accuracy has a small drop (seen in Figure 8(b). (3) TCA + 1NN, TCA + SVM, JDA + 1NN, and JDA + SVM, as two-stage feature transfer classifier (i.e., first feature extraction and then classification), is little weaker because their feature extraction and classification process is separated and cannot be unified into a unified optimization framework.

### 4.3. Parameter Analysis

To evaluate the performance variations of our TELM-OWA with the target domain labeled sample ratio (*g*), the number of hidden layer nodes (*L*) and balance parameters *λ*, *γ*, *θ*, we conduct the experiments on the 4 datasets like org versus people, MSRC versus VOC, MNIST versus USPS, A versus D and the results are shown in Figures 8(a)–8(e). The following can be seen: (1) with the increase of the number of target labeled samples for training ELM, the accuracy of TELM-OWA is increasing, as shown in Figure 8(a). It can be known that when the target domain label sample is small, the source domain knowledge can help the target domain task. With target labeled sample increasing, the trained model better fits target data and has higher accuracy. (2) As shown in Figure 8(b), the accuracy of TELM-OWA increases with the number of hidden layer node on the 4 datasets. This verifies that a huge amount of hidden nodes are beneficial because they may force the ELM network to behave better on output function approximation. (3) In Figure 8(c), with the gradual increase of *λ*, the accuracy increases first and then little decreases. When *λ* is too small, the helpful information from source domain is underutilized leading to the low performance. When *λ* is too large, the trained model overfits the source domain samples, resulting in performance degradation. TELM-OWA achieves a good result when *λ* ∈ [10,100]. Dataset org versus people is robust to changes in parameter *λ*. (4) In Figure 8(d), the accuracy exhibits a little rising and then declining tendency with increase of *γ*, in which better accuracy is obtained when *γ* ∈ [10,  100]. When *γ* is small, the performance is a little low because *β*_*S*_ is far from *β*_*T*_. When *γ* is too large, ‖*β*_*T*_ − *β*_*a*_‖^2^ will reduce the influence of the empirical risk error of labeled sample from source and target domains and the accuracy will degrade. (5) As shown in Figure 8(e), the accuracy increases first and then decreases with the increasing of the parameters *θ* which control the quality of *β*_*S*_ and achieves better classification results when *θ* ∈ [10^−4^, 10^−3^].

## 5. Conclusion

To solve the problem of the performance degradation of the traditional Extreme Learning Machine algorithm in the case of a small number of reliable training samples, in this paper, we propose TELM-OWA which is an Extreme Learning Machine with the ability of knowledge transfer. It reduces the distribution difference across domains by aligning the ELM output weight matrix between domains and introducing the approximation between the interdomain ELM output weight matrices to the objective function. Moreover, the objective function is transformed to the standard ELM form to solve. Many experiments were designed to compare our proposed algorithm with other related algorithms, and the results show that TELM-OWA has higher accuracy and better generalization performance.

TELM-OWA still has some limitations: (1) it still needs some labeled samples in the target domain, and it is not suitable for the supervised transfer learning environment. (2) It reduces the distribution difference across domains by aligning the ELM output weight matrix between domains and ignore the overall distribution differences in the output layer, in which the divergence of statistical distributions between the source and target domains still is different due to variance among each dimension. (3) Its shallow architectures lead to failure to find higher-level representations and thus can potentially capture relevant higher-level abstractions.

As a result, the following research focuses on the following three aspects to improve TELM-OWA: firstly, reliable samples selection is introduced for unsupervised transfer learning. Secondly, the effectiveness of knowledge transfer is further promoted by aligning the ELM output weight matrix and minimizing the divergence of statistical distributions together. Thirdly, as is similar to deep learning, TELM-OWA is improved by stacking it into a deep structure model for extracting deep feature.

## Figures and Tables

**Algorithm 1:**
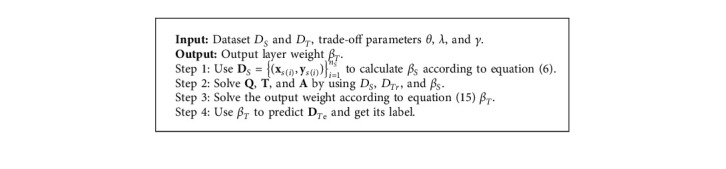
TELM-OWA

**Table 1:** Description of image and text datasets.

Dataset		Type of data	Number of samples	Dimension	Class	Contains subsets
USPS		Digit	1,800	256	10	USPS
MNIST		Digit	2,000	256	10	MNIST
MSRC		Object	1,269	240	18	MSRC
VOC2007		Object	1,530	240	20	VOC
Caltech-256		Object	1,123	800	10	Caltech
Office	AMAZON	Object	958	800	10	AMAZON
	Webcam	Object	295	800	10	Webcam
	DSLR	Object	157	800	10	DSLR
Reuters-21578:	Orgs	Text	1,237	4,771	Binary	Orgs
	People	Text	1,208	4,771	Binary	People
	Place	Text	1,016	4,771	Binary	Place

**Table 2:** Accuracy of different algorithms on image and text datasets.

Dataset	Nontransfer learning algorithm				Transfer learning algorithm							
	1NN	SVM	ELM	SSELM	TCA + 1NN	TCA + SVM	JDA + 1NN	JDA + SVM	DAELM_S	DAELM_T	ARRLS	TELM-OWA
USPS vs. MNIST	83.41	78.60	88.52	90.95	85.22	83.66	84.78	83.66	88.40	90.77	85.34	91.95
MNIST vs. USPS	91.07	84.76	94.25	94.32	90.24	83.86	87.33	84.70	92.59	93.56	91.97	94.18
*Average*	87.24	81.68	91.39	92.64	87.73	83.76	86.05	84.18	90.49	92.16	88.65	93.07

MSRC vs. VOC	40.31	42.75	46.74	44.38	34.20	46.82	34.85	48.13	46.74	40.15	45.11	50.16
VOC vs. MSRC	61.46	51.72	75.52	78.86	68.63	70.40	67.85	72.34	76.20	80.14	68.63	85.05
*Average*	50.88	47.24	61.13	61.62	51.42	58.61	51.35	60.23	61.47	60.14	56.87	67.61

C⟶A (1)	32.30	60.83	61.22	61.22	56.03	63.16	56.16	65.50	62.00	59.79	60.70	70.04
C⟶W (2)	31.80	48.12	56.07	53.97	61.09	60.67	59.83	59.83	61.92	69.87	53.14	68.20
C⟶D (3)	26.15	44.62	46.92	43.08	47.69	52.31	48.46	53.85	50.77	64.62	43.08	55.38
A⟶C (4)	27.27	50.00	49.33	45.57	45.90	51.77	43.90	52.66	48.67	53.88	46.67	50.89\
A⟶W (5)	40.59	57.32	49.37	46.44	59.83	54.39	56.07	54.81	52.72	64.85	47.28	69.04
A⟶D (6)	26.92	40.00	39.23	43.08	36.92	46.92	50.00	46.15	46.92	60.00	42.31	53.08
W⟶C (7)	26.94	49.11	43.79	44.24	42.57	49.56	40.35	48.78	44.68	54.77	43.46	45.12
W⟶A (8)	39.95	61.35	56.68	53.57	57.20	63.68	58.75	66.54	60.96	64.07	55.64	62.78
W⟶D (9)	63.85	89.23	81.54	83.08	91.54	90.00	90.77	86.15	77.69	64.62	84.62	81.54
D⟶C (10)	27.16	48.56	47.67	47.23	43.24	51.55	43.90	51.22	47.01	56.32	46.23	45.01
D⟶A (11)	42.80	64.85	60.18	57.98	60.05	68.48	57.98	68.48	62.26	63.55	62.13	61.87
D⟶W (12)	66.11	83.68	82.43	85.36	92.47	87.03	89.12	89.54	81.59	60.67	91.63	88.28
*Average*	37.65	58.14	56.20	55.40	57.88	61.63	57.94	61.96	58.10	61.42	56.41	62.60

Orgs vs. people (1)	80.04	83.04	82.73	84.49	75.39	77.25	75.08	81.28	82.94	85.01	83.66	87.28
People vs. orgs (2)	81.94	85.57	85.67	87.59	75.68	77.60	74.37	84.36	88.09	84.36	87.39	88.50
Orgs vs. place (3)	74.85	79.16	74.97	77.25	69.58	71.98	72.57	75.09	79.64	79.76	78.56	84.67
Place vs. orgs (4)	75.55	78.38	79.12	81.94	70.39	69.04	70.39	78.26	82.06	85.63	80.34	88.45
People vs. place (5)	64.62	70.30	64.73	72.74	60.79	60.32	61.60	65.55	74.48	79.47	72.27	87.12
Place vs. people (6)	60.60	61.18	61.88	65.24	58.29	61.80	58.63	67.01	69.87	72.54	62.69	78.33
*Average*	72.93	76.27	74.85	78.21	68.35	69.66	68.77	75.26	79.51	81.13	77.49	85.73

*Total average*	52.99	64.23	64.93	65.57	62.86	65.56	62.85	67.45	67.19	69.47	65.13	72.13

**Table 3:** Consuming time of different approaches on MNIST versus USPS.

Algorithm	1NN	SVM	ELM	SSELM	TCA + 1NN	TCA + SVM	JDA + 1NN	JDA + SVM	DAELM_S	DAELM_T	ARRLS	TELM-OWA
Time (s)	0.55	8.79	0.37	3.49	4.72	5.47	48.32	53.6	0.81	0.64	2.08	3.7

## Data Availability

To verify the validity of TELM-OWA, four different datasets, Office + Caltech object recognition, USPS and MNIST digital handwriting, MSRC and VOC2007 object recognition, and Reuters-21578 text dataset, are used for classification experiments. (1) https://github.com/jindongwang/transferlearning/blob/master/data/dataset.md; (2) https://www.cse.ust.hk/TL/index.html; and (3) http://ise.thss.tsinghua.edu.cn/∼mlong/publications.html. MSRC and VOC2007 object recognition datasets are released in the paper named “Transfer Joint Matching for Unsupervised Domain Adaptation”.
